# The relationship between students’ perception of the educational environment and their subjective happiness

**DOI:** 10.1186/s12909-019-1851-0

**Published:** 2019-11-08

**Authors:** Dong-Mi Yoo, Do-Hwan Kim

**Affiliations:** 1 Department of Medical Education, College of Medicine, The Catholic University of Korea, Banpo-daero 222, Seocho-gu, Seoul, 06591 Republic of Korea; 2 Department of Medical Education, Hanyang University College of Medicine, 222 Wangsimni-ro, Seongdong-gu, Seoul, 04763 Republic of Korea

**Keywords:** Dundee ready educational environment measure, Educational environment, Undergraduate medical education, Happiness, Academic achievement

## Abstract

**Background:**

Happiness, a subjective judgment about one’s quality of life, is influenced by environmental factors and should be considered as an important goal of medical education, which should support each learner’s development as a person as well as a professional. However, although several studies have reported on the correlation between Dundee Ready Educational Environment Measure (DREEM) scores and students’ academic achievement, few have investigated the relationship between DREEM scores and students’ subjective happiness. This study examined different perceptions of the educational environment between phases of the curriculum and determined which DREEM subscales affect the overall level of happiness.

**Methods:**

We used the Korean version of the DREEM questionnaire and a single item measure of happiness on a scale of 0 to 10. First we analyzed student perceptions of the educational environment according to their demographic characteristics using independent sample t-tests and one-way analysis of variance. A multiple regression analysis was performed to reveal which subscales affect the overall level of happiness while controlling for grade point average (GPA) and other demographic characteristics.

**Results:**

The subjects were 239 medical school students across all stages of the curriculum. The students’ overall perception was more positive for the educational environment during Phase 3 (clerkship) than Phase 1 (pre-medical). Among the DREEM subscales, this difference was especially prominent in Students’ Perception of Learning and Students’ Academic Self-Perceptions. In contrast, no difference in the subjective perception of happiness was found between phases. The effect of GPA on happiness became insignificant under the control of other variables, but the influence of the Students’ Social Self-Perceptions (SSSP) subscale remained significant.

**Conclusions:**

The students’ overall perception of the educational environment was more positive during the clerkship period than in the pre-medical period. Based on our finding that the SSSP correlates significantly with subjective happiness, we suggest that institutions promote not only students’ academic development but also their happiness by fostering an appropriate educational environment.

## Background

In the era of competency-based medical education (CBME), fostering a “competent doctor” who has integrated knowledge, skills, and attitudes aligned with the health needs of the population is an important goal of undergraduate medical education (UME) [1, 2]. However, despite the advantages of CBME, several concerns have been raised recently about its negative effects on individual learners, such as burnout among medical students [3]. One possible reason for those effects is that the newly emphasized competencies act as drivers of stress and burnout because they’ve been added to an already overcrowded curriculum [4]. The alarming rate of burnout and depressive symptoms among practicing physicians and medical students, which continues to increase, indicates that the problem has not been effectively addressed [5–7], although CBME might not be its only cause.

Thus, it has been argued that fostering so-called “good doctors” who have adequate knowledge, skills, and attitudes cannot be the sole aim of medical education [8]. Situated learning theory argues that merely acquiring medical knowledge and skills is insufficient for the formation of an identity [9, 10]. Given that all students manage multiple identities [11], medical education has to help learners integrate their personal and professional development [4, 12]. But personal development often holds only a secondary position in the curriculum [13] or competency domain [14]. In this context, it has been claimed that medical education should work to generate doctors who are both good and happy, rather than one to the exclusion of the other [15].

But what does it mean to be a “happy doctor” beyond mere rhetoric? First, existing studies of happiness generally define it as a “global evaluation of [an] individual’s life quality according to their own criteria” [16]. It conceptually overlaps with quality of life (QOL) and subjective well-being, sharing physical, psychological, social, and environmental aspects as common components [16, 17]. For medical students, Dunn et al. explained that well-being is an outcome that combines personal factors being represented as a reservoir and environmental factors expressed as positive and negative input [18]. They also indicated that the influence of a particular input is not fixed; the same stressor can be perceived differently depending on a learner’s past experience or current state [18].

If happiness can be understood as a subjective judgment influenced by environmental factors, then medical students’ happiness will be affected by the educational environment, which makes up the major physical, temporal, and relational portions of their lives. More important, similar to the varying effects of inputs and stressors, it could be that students’ perceptions of the educational environment, rather than the environment itself, actually influence their happiness [4]. This possibility is consistent with the constructivist view that values learners’ autonomous and subjective constructions in learning based on their existing experiences, perceptions, and knowledge [10]. Indeed, the perception of an educational environment affects not only students’ academic achievement [19] but also the formation of their professional identity [20]. Therefore, researchers in the field of health-professions education have made considerable efforts to measure learners’ perceptions of the educational environment. Among the variety of available tools, the Dundee Ready Educational Environment Measure (DREEM) inventory has gained wide popularity and been deemed the most suitable tool for UME settings [21].

Numerous reports using DREEM have been made since its development [22]. However, important research areas remain untouched by existing research. First, according to a recently published systematic review [23], 52% of published research using DREEM originated in Asia while 40% originated in the Middle East. Although the majority of DREEM publications were from Asia, few studies using DREEM have been published from East Asian countries including China, Japan and South Korea, which have many medical schools [24] and that limits the applicability and generalizability of previous findings because the Asian sub-regions differ in their social development, health care needs, and educational traditions [25–27]. Furthermore, the specific structures used in medical education generally and the specifics of each educational stage vary widely from country to country [28]. For instance, the UME system in South Korea is distinguished from that of China or Japan, as well as from other parts of the globe, in using undergraduate-entry programs, and its first two years of pre-medical curriculum are fairly separate from the following four-year medical curriculum [29].

Second, the relationship between DREEM scores and possible correlates beyond academic achievement needs further examination [23]. Indeed, no previous studies have used the term “happiness,” and only a few studies have reported on the correlation between DREEM scores and well-being, using constructs such as QOL and resilience, and those which have been conducted had their own limitations. For example, a possible association between high DREEM scores and QOL was suggested by two studies conducted in a single country [30, 31], and although they successfully controlled for sex, age, and year, GPA, a main cause of mental distress among students [32], was not included as an independent variable. Similarly, another study that showed an association between the Medical Student Well-Being Index and academic self-perceptions, a subscale of DREEM, also failed to use appropriate objective data for academic performance and included atypical subjects, such as international medical students [33].

Assuming that the educational environment influences the subjective happiness of medical students at all, researchers need to be able to specify which factors in that environment are relevant. Therefore, we first sampled and examined overall DREEM scores from students across all stages of the curriculum at a medical school in South Korea. Subsequently, to determine which subscales of DREEM affected the overall level of happiness, we investigated the relationship between the subscales and happiness while controlling for GPA and other demographic characteristics.

## Methods

### Study design

The study is cross-sectional and follows the pragmatic research tradition. Regarding terminology, although two terms — educational environment and learning environment — are often used interchangeably, we use only educational environment in this study because it can cover “everything that is happening in the medical school” [34]. Some environmental elements in medical school could be more relevant to subjective happiness than to learning and vice versa.

### Setting

This study was conducted at the Eulji University School of Medicine (EUSOM), located in the city of Daejeon, South Korea. EUSOM is a six-year private medical school, and its curriculum is largely divided into three stages. Phase 1 corresponds to medical school year 1 (M1) and medical school year 2 (M2) and consists mainly of basic science courses, such as biology, chemistry, and physics. Social sciences and humanities, commonly considered as pre-medical requirements [35], are also taught during this phase. Phase 2 corresponds to M3 and M4, and most courses in this phase use an organ-based integrated curriculum to teach basic and clinical science. Phase 3 comprises M5 and M6 and uses a curriculum focused on a clinical clerkship at the affiliated teaching hospital. Each year, the number of students per year level has remained around 45; in 2018, the total number of students was 281, with 47 M1 students, 51 M2 students, 51 M3 students, 43 M4 students, 45 M5 students, and 44 M6 students.

### Data collection

The survey was conducted from May to July, the latter half of the first semester of 2018. To collect data comprehensively from all M1 to M6 students, the survey schedule was arranged based on the curricular schedule for each cohort. Prior to data collection, the purpose of the survey was explained, and written information was provided. Each student could decide whether to take part in the survey. The Institutional Review Board waived the need for explicit consent from participants by considering return of the survey as consent to participate. Of the total 281 students, 243 students initially submitted responses. If a response omitted only one item, it was included, and the missing item was replaced with the average value of the remaining items. However, if a response omitted more than one item, it was excluded and considered an incomplete response. To collect demographic information about the participants, the survey asked for the students’ identification (ID) numbers. For those who provided their ID number, we collected data such as their age, sex, and GPA from the institutional database.

### Instruments

#### Korean version of the Dundee ready educational environment measure

The Korean version of the DREEM survey was identical to the one used by the Korean Society for Medical Education in 2013 for a nationwide analysis of all medical schools in South Korea [36]. The survey contains 50 questions, of which nine are reverse-scored. Each item is measured on a five-point Likert scale from 0 (strongly disagree) to 4 (strongly agree). These 50 items can be analyzed on three levels: overall score, five subscales, and individual items. An open-ended question included in the original DREEM inventory was not included in this study.

In terms of subscales, the overall survey contains 12 questions reflecting Students’ Perception of Learning (SPL), 11 questions reflecting Students’ Perception of Teaching (SPT), eight questions about Students’ Academic Self-Perceptions (SPP), 12 questions about Students’ Perceptions of Atmosphere (SPA), and seven questions about Students’ Social Self-Perceptions (SSSP). In this study, the three items that require at least some form of direct experience in a clinical setting, items number 6 (SPT), 11 (SPA), and 18 (SPT), were asked only of Phase 3 students because the EUSOM curriculum structure makes it difficult for Phase 1 and 2 students to provide valid responses to those questions.

#### Single-item measure of happiness

In this study, we used a single-item measure of happiness: “To what extent do you think you are living a happy life?” with an 11-point scale (0: not at all – 10: a great deal) based on a previous study that showed high temporal reliability and concurrent, convergent, and divergent validity of happiness measured by a single item [37]. Similarly, it has been reported that happiness can be validly and reliably measured using a single-item measure [17], and evidence shows that happiness, subjective well-being, and QOL are interchangeable with one another in terms of their construct [16]. Furthermore, in terms of response rates, studies have confirmed that shorter surveys generally provide better results [38]. Because the DREEM already requires answers to as many as 50 questions, we aimed to increase the compliance of participants by reducing the additional burden and using a single-item measure for happiness.

### Data analysis

We included 239 valid responses in our analysis, after excluding four incomplete responses. In general, the DREEM scores are reported as the summation of each item. However, because students in Phases 1, 2, and 3 answered 47, 47, and 50 items, respectively, we used the mean score of items to accurately reflect the differences in the maximum total scores possible for each phase.

The overall and subscale scores were analyzed using parametric statistics. Despite controversy about the appropriateness of treating a Likert response as a number, it has been strongly argued that the summed score of many items is suitable for use with parametric methods as interval data [39]. The distribution of data was assessed using Kolmogorov-Smirnov tests and boxplots. Cronbach’s alpha was then calculated to check the internal consistency of each subscale. We did not use Cronbach’s alpha for the overall DREEM score because it could inappropriately inflate the alpha value [40].

For the DREEM scores and subjective happiness measurements, we analyzed differences between the means using independent sample t-tests or one-way analysis of variance (ANOVA). To reduce the risk of type 1 errors during multiple pairwise comparisons, we used Tukey’s HSD test for post-hoc comparison of the ANOVA results [41, 42]. Pearson’s correlation coefficients were calculated to examine the univariate relationships between variables. A multiple regression analysis was performed to examine the influence of the five DREEM subscales and GPA while controlling for demographic factors such as age and sex. *P*-values of less than 0.05 were considered statistically significant.

To interpret the strength of the Pearson correlation coefficient, we followed Evans’ classification (0.00–0.19, very weak; 0.20–0.39, weak; 0.40–0.59, moderate; 0.60–0.79, strong) [43]. Eta squared was used to calculate the effect size and was interpreted based on Cohen’s recommendation (0.01, small effect; 0.06, medium effect; 0.14, large effect) [44]. For all statistical analyses, we used IBM SPSS Statistics for Windows software (version 20; IBM Corp., Armonk, NY, USA).

## Results

### Demographic characteristics

Table 1 displays the demographic characteristics of the respondents. Overall, 243 responses were received from the total of 281 students; 239 of those responses were considered valid, for an overall response rate of 85.1%. The 229 students (81.5%) who provided their student ID number with the survey and 52 students who did not respond to the survey showed no significant difference in gender (*p* = 0.870) or previous GPA (*p* = 0.994). However, the two groups did differ significantly in age (*p* = 0.025 in independent sample t-test) and phase (*p* = 0.003 by Pearson’s chi square), which correlated significantly with age.

**Table 1:** Demographic characteristic of the responders

Variables		Phase 1	Phase 2	Phase 3	Total respondents
Valid Respondents Proportion (frequency)		37.7%(90)	33.1%(79)	29.3%(70)	100%(239)
Response rate		91.8%	84.0%	78.7%	85.1%
Age (yr)^a)^ Mean (SD)		20.7 (1.60) (range: 19–26)	23.3 (2.21) (range: 21–34)	25.3 (2.13) (range: 23–34)	22.9 (2.78) (range: 19–34)
Gender ^a)^ Proportion (frequency)	Male	68.9%(62)	66.1%(41)	64.3%(45)	61.9%(148)
	Female	31.1%(28)	33.9%(21)	35.7%(25)	31.0%(74)
Year Proportion (frequency)		Year 1: 52.2%(47), Year 2: 47.8%(43)	Year 3: 50.6%(40), Year 4: 49.4%(39)	Year 5: 41.4%(29), Year 6: 58.6%(41)	
Previous Year GPA^a), c)^ Mean (SD)		3.20 (0.69) ^b)^ (range: 1.48–4.44)	3.22 (0.64) (range: 2.10–4.38)	3.23 (0.62) (range: 2.06–4.43)	3.22 (0.64) (range: 1.48–4.44)

a) Fourteen students who participated in the survey but did not disclose their student ID numbers were excluded; b) Year 1 students who entered in 2018 were excluded due to their lack of previous GPA data at the time of analysis; c) EUSOM follows the A–F grading system (A + =4.5, A = 4, B + =3.5, B = 3, C + =2.5, C = 2, D + =1.5, D = 1, and F = 0). An A+ in all subjects produces a maximum GPA of 4.5, and an F in all subjects produces a minimum GPA of 0

*Abbreviation*: *SD* Standard Deviation, *GPA* Grade Point Average

Of the 239 valid respondents, 61.9% were male, and the sex balance was similar throughout the phases. Phase 1, Phase 2, and Phase 3 accounted for 37.7, 33.1, and 29.3% of respondents, respectively. The average age of students differed significantly by phase (*p* < 0.001) but not by sex (*p* = 0.779) or the previous year’s GPA (*p* = 0.950).

### Cross-sectional analysis of educational environment and subjective happiness

The DREEM and happiness scores were first investigated with regard to their relationships with demographic factors (Table 2). The Cronbach’s alphas for the SPL, SPT, SASP, SPA, and SSSP were 0.783, 0.753, 0.579, 0.745, and 0.573, respectively. The DREEM scores did not differ by sex but those of Phase 3 students were significantly higher than those of Phase 1 students (*p* = 0.007). The DREEM scores also correlated significantly with age and GPA, but with weak or very weak strengths of approximately 0.2. Only GPAs correlated significantly with subjective happiness, and that connection was weak; no other demographic variables demonstrated any significant association with subjective happiness.

**Table 2:** DREEM and happiness scores by demographic characteristics (univariate analysis)

Variables		SPL	SPT	SASP	SPA	SSSP	DREEM Score	Subjective Happiness
All participants Mean (SD)		2.18 (0.52)	2.59 (0.49)	2.37 (0.71)	2.31 (0.49)	2.31 (0.51)	2.34 (0.44)	6.61 (1.99)
Age	Correlation^a)^	0.224	0.195	0.332	0.092	0.042	0.226	−0.003
	*p*-value	0.001	0.003	< 0.001	0.17	0.529	< 0.001	0.97
Gender Mean (SD)	Male	2.18 (0.51)	2.58 (0.49)	2.33 (0.61)	2.37 (0.50)	2.35 (0.46)	2.35 (0.43)	6.71 (2.03)
	Female	2.17 (0.49)	2.63 (0.49)	2.37 (0.52)	2.24 (0.46)	2.26 (0.58)	2.33 (0.41)	6.61 (1.90)
	*p*-value^b)^	0.912	0.439	0.627	0.066	0.26	0.67	0.71
Phase Mean (SD)	1	2.08 (0.57)	2.52 (0.54)	2.07 (0.58)	2.28 (0.50)	2.31 (0.52)	2.24 (0.45)	6.68 (2.14)
	2	2.19 (0.51)	2.64 (0.50)	2.45 (0.89)	2.30 (0.52)	2.24 (0.54)	2.35 (0.46)	6.33 (2.06)
	3	2.30 (0.42)	2.63 (0.41)	2.66 (0.43)	2.37 (0.45)	2.38 (0.46)	2.46 (0.36)	6.83 (1.65)
	F(p-value) ^c)^	3.599 (0.029)	1.579 (0.208)	16.723 (< 0.001)	0.739 (0.479)	1.432 (0.241)	5.087 (0.007)	1.233 (0.293)
	Post-hoc ^c)^	1 < 3		1 < 2,3			1 < 3	
	Eta-squared	0.030		0.124			0.041	
Previous Year GPA	Correlation ^a)^	0.095	0.054	0.216	0.163	0.167	0.167	0.197
	p-value	0.209	0.477	0.004	0.031	0.027	0.027	0.010

a) Pearson correlation; b) Independent sample t-test; c) Tukey’s HSD test was used to analyze data with equal variance

*Abbreviation*: *SASP* Students’ Academic Self-Perceptions, *SPA* Students’ Perceptions of Atmosphere, *SPL* Students’ Perception of Learning, *SPT* Students’ Perception of Teaching, *SSSP* Students’ Social Self-Perceptions

Table 3 shows the correlation between happiness and other variables. The total DREEM scores correlated significantly with happiness, regardless of phase (Phases 1, 2, 3, and all participants), but GPA lost its significance when analyzed by phase. When the DREEM was divided into subscales, the SSSP and SPA demonstrated relatively strong correlations, with values of 0.560 and 0.423, respectively. In particular, the correlations between the SSSP and happiness scores were above 0.5 in all phases.

**Table 3:** Correlations between happiness and other variables

		SPL	SPT	SASP	SPA	SSSP	DREEM Score	Previous Year GPA
All participants (229)	Correlation^**a)**^	0.221	0.100	0.247	0.423	0.560	0.363	0.197
	p-value	0.001	0.132	0.000	0.000	0.000	0.000	0.010
Phase 1 (87)	Correlation^**a)**^	0.229	−0.004	0.391	0.529	0.544	0.392	0.136
	p-value	0.033	0.973	0.000	0.000	0.000	0.000	0.396
Phase 2 (76)	Correlation^**a)**^	0.201	0.250	0.172	0.351	0.574	0.355	0.226
	p-value	0.082	0.030	0.137	0.002	0.000	0.002	0.077
Phase 3 (66)	Correlation^**a)**^	0.245	0.103	0.283	0.344	0.550	0.351	0.221
	*p*-value	0.047	0.412	0.022	0.005	0.000	0.004	0.074

a) Pearson correlation

*Abbreviation*: *SASP* Students’ Academic Self-Perceptions; *SPA* Students’ Perceptions of Atmosphere, *SPL* Students’ Perception of Learning, *SPT* Students’ Perception of Teaching; *SSSP* Students’ Social Self-Perceptions

### Factors associated with subjective happiness

The multiple regression analysis produced a significant regression eq. (F (9, 158) = 17.892, *p* < 0.001) with an R^2^ of 0.505. When demographic variables were controlled, SSSP and SPT were the significant factors affecting happiness among the DREEM subscales, with SSSP (β = 0.628, p < 0.001) predicting happiness more powerfully than SPT (β = − 0.131, *p* < 0.027) (Table 4). Students’ demographic characteristics and the other DREEM subscales — SPL, SASP, and SPA — were not significant predictors of happiness.

**Table 4:** Multiple regression analysis

Dependent Variable	Independent Variables	Unstandardized coefficient		Standardized coefficient	t	*p*-value	Adj R^2^ (F)
		B	Standard error	β			
Subjective Happiness	(Constant)	−2.097	1.557		−1.347	0.180	0.477 (17.892)
	Gender (Female)	0.350	0.257	0.084	1.365	0.174	
	Age	0.107	0.059	0.136	1.821	0.070	
	Phase	−0.074	0.206	−0.029	−0.361	0.719	
	Previous Year GPA	0.257	0.189	0.082	1.361	0.175	
	SPL	−0.557	0.393	−0.131	−1.419	0.158	
	SPT	−0.819	0.366	−0.183	−2.240	0.027	
	SASP	0.636	0.334	0.181	1.905	0.059	
	SPA	0.528	0.397	0.133	1.331	0.185	
	SSSP	2.394	0.300	0.628	7.993	0.000	

*Abbreviation*: *SASP* Students’ Academic Self-Perceptions, *SPA* Students’ Perceptions of Atmosphere, *SPL* Students’ Perception of Learning, *SPT* Students’ Perception of Teaching, *SSSP* Students’ Social Self-Perceptions

## Discussion

In this study, we used the DREEM to examine the environment of a medical school and understand its relationship with the subjective happiness of students. The overall perceptions of the educational environment were more positive during the clerkship period than in the pre-medical period, and this tendency was especially prominent in the SPL and SASP subscales. By contrast, subjective happiness did not differ significantly between phases and had a stronger correlation with SPA and SSSP than with the other subscales. When other variables (sex, age, phase, and GPA) were controlled, only the influence of SSSP on subjective happiness remained significantly positive.

### Overall and subscale DREEM scores

When we converted our mean DREEM scores by multiplying them by 50, which is the total number of items, to make them comparable to previous studies that reported the summation of individual items, 112, 117.5, and 123 points were obtained for Phases 1, 2, and 3, respectively. According to the suggested guideline for interpretation [45], those converted DREEM scores from students in all three phases at EUSOM were categorized as “more positive than negative.” However, that range (101–150 points) contains more than 80% of the DREEM scores reported from various countries [23]. According to a nationwide cross-sectional study in Japan, for example, 77 out of 80 medical schools were in the “more positive than negative” category [46].

Although the scores from all three phase fit into the same category, an increasing tendency was maintained through the phases from 1 to 3. This is in contrast to previous studies, which reported that lower DREEM scores were related to seniority [23] or found a u-shaped pattern with high scores at the beginning and end and low scores in the middle [40]. It is thus worth examining why the phase 1 students in this study gave significantly lower scores than the phase 2 and 3 students.

First, the characteristics of the EUSOM curriculum in phases 2 and 3 might have produced the difference. According to previous studies, an integrated curriculum such as that used in phase 2 can induce more positive perceptions than a curriculum organized by discipline, such as that used in phase 1 [47]. Furthermore, phase 3 emphasizes each student’s authentic engagement as a member of a clinical team in a hospital setting, whereas phases 1 and 2 mainly involve listening to didactic lectures in a classroom setting. We assume that those differences in the level of student participation improved the SPL and SASP subscale scores in phase 3 by positively influencing on that phase’s study-centeredness of education and academic self-efficacy of students [48]. Indeed, student perceptions of their level of engagement, though not measured in DREEM, have been suggested as a key factor in determining the educational environment [49].

Second, the current undergraduate medical education system in South Korea could also explain the improvement in DREEM scores from phase 1 to phase 3. The system is a unique model introduced during the colonization era of the early twentieth century [50], with phase 1 (pre-medical) operating in isolation from the following four years of medical education. As a result, it has long been argued that students suffer from deteriorating academic motivation, study habits, and professional identity formation during phase 1 [29]. The lower SPL and SASP scores, which are the subscales most directly related to academic and professional development, seem to reflect this current state of pre-medical education, which also contributed greatly to the lower overall DREEM scores from phase 1 students.

### Subjective happiness and DREEM scores

Interestingly, in contrast to the DREEM scores, subjective happiness did not differ between phases. If the significant difference in the DREEM scores reflects differences between the pre-medical and clinical phases, this result suggests that more positive perceptions of the educational environment do not necessarily guarantee happier students. In other words, although DREEM scores might reflect professional development into the “good doctors” expected by society, medical schools should also pay keen attention to students’ personal development to ensure that they grow into “happy doctors” as well.

In this regard, it is worth mentioning that among the subscales, only SSSP had a significant positive influence on subjective happiness when other variables were controlled in the multiple regression analysis (Table 4). To put it another way, SPL and SASP, which are more directly related to schoolwork, exerted only a minimal effect on subjective happiness. This finding — social self-perception plays a primary role in the subjective happiness of medical students — is consistent with previous studies that highlighted the importance of social support in the general population [51].

However, that finding contradicts a study from a Brazilian medical school that found that all subscale scores and the total DREEM score were associated with QOL [31], which is regarded as interchangeable with happiness [16]. This difference could result from differences in the criteria used to judge happiness in the two cultures [52].]. Researchers have shown that East Asian cultures tend to focus on interpersonal aspects in the perception of happiness [53, 54]. Similarly, in a collectivism-oriented culture, the importance of an individual’s social acceptance tends to be prominent [55] and could explain why the SSSP and subjective happiness had the strongest correlation in our results.

Therefore, setting the pursuit of academic development as the solitary goal of medical education might not be desirable, particularly if it is weighted heavily toward individual achievement, because improving subjective well-being through higher academic achievement is uncertain; we did not find that the contribution of GPA to subjective happiness was significant in our multiple regression. Instead, the stress that comes from acquiring a higher GPA can trigger burnout [18], which could hinder the development of professional attitudes and values [56]. The limited connection between a high GPA and happiness might also be explained by EUSOM’s use of the A–F grading system, which has been shown to cause distress and anxiety regardless of one’s level of achievement [4].

In summary, as the title of one Korean film put it, “Happiness Does Not Come in Grades (Woo-suk Kang, 1989).” Rather, our findings indicate that social relationships are a more important factor. Our findings clearly suggest that students’ subjective happiness levels and their perceptions of the educational environment cannot be easily or straightforwardly changed by aiming for higher academic achievement.

### Limitations

This study had several limitations. First, its generalizability could be limited by its cross-sectional design and its use of data from a single university. The general characteristics of EUSOM students, as well as the specific institutional and cultural context, might have affected the results of this study. Although our response rate was 85.1%, it is possible that the non-responders in phase 3 had a more negative perception of the educational environment than the responders, which is why they chose not to participate in the survey. Second, in terms of the collected data, the Cronbach’s alphas of SASP and SSSP, 0.579 and 0.573, respectively, were relatively low. However, it is well known that Cronbach’s alpha is sensitive to the number of items [57], and these two subscales were composed of eight and seven items, respectively, which is fewer than the other subscales. Third, as a quantitative measure, the DREEM has the clear advantage of enabling intra- and inter-medical school comparisons, but the weakness of the instrument itself also needs to be considered. Concerns have been raised about the psychometric robustness of the DREEM, such as its internal consistency and construct validity, as well as the need to revise items [58, 59]. Our findings of lower Cronbach’s alpha values for the SASP and SSSP subscales could be partly attributable to this psychometric weakness of the DREEM. In addition, its insufficient validity evidence has been pointed out as a weakness, especially considering its popularity [60]. Fourth, this research could not determine causal relationships. For example, in the relationship between DREEM scores and happiness, either side could be the cause of the other, at least partly, or both could be caused by a third, unstudied variable.

### Implications for medical educators and future research

This study has the following implications for educational practice and future research. First, based on the overall DREEM scores, this study suggests that improving the educational environment for junior students, rather than seniors, needs to be a priority. This period should be given particular consideration because the pre-medical period comprises a significant portion of UME, and the formation of a professional identity starts at the beginning of medical education. Moreover, it has been argued that junior students in medical schools tend to have higher expectations for educational responsibility than senior students [61], which might have contributed to the lower DREEM scores.

For further study, both global and interdisciplinary research is needed to deepen understanding about longitudinal changes in overall DREEM scores across educational years. Most of all, the upward sloping tendency in the DREEM scores that we found might not be a representative pattern. The previous literature has shown mixed results, including a downward slope [23] that suggests that junior students gave higher ratings than seniors and a u-shaped pattern in which negative perceptions got stronger in the middle of the curriculum [40]. Therefore, it might be premature to conclude that any universal or prescribed tendency exists in how students in the health professions change their perceptions throughout the curriculum. A comparison study might be able to identify the main causes for the differences found between institutions or disciplines.

At the local and regional level, studies are needed to explore the possible influence of the East Asian, national, and institutional contexts, possibly by using a mixed method or qualitative approach that can sidestep the limitations of the quantitative approach [62]. Looking at the individual items included in each subscale might also be useful; this study analyzed survey results only at the subscale and overall level. To interpret at the item level, it has been recommended that researchers focus on the ratio of three categories — strongly disagree/disagree, unsure, and strongly agree/agree — rather than the average scores because a skewed or bimodal distribution often occurs at the item level [63].

Second, the relationships among the DREEM scores, GPA, and happiness suggest that investment in and support for students’ achievements need to be balanced with investments to promote their positive social perceptions and relationships. In the short run, it is well known that social relationships are very closely related to professional identity formation in medical schools [64]. In the long term, skills in maintaining supportive relationships both personally and professionally are critical to prevent burnout among physicians [65].

To attain a balance between academic achievement and social relationships, medical schools need to shift their view of learning from an “acquisition” model centered on individuals and independence to a “participation” model that emphasizes social relationships and interactions [66]. That change is consistent with situated learning theory, a perspective of medical education that considers learning to be “inextricably tied to its context and to the social relations” [10]. In practice, one recommended approach in UME could be the introduction of a pass–fail grading system to promote a collaborative environment among students. The fact that such a grading system would facilitate students’ well-being without decreasing their academic performance [67] suggests that cultivating doctors who are both happy and competent is not an impossible goal.

For future study, exploring variables that correlate significantly with DREEM scores would be an important task. Further understanding the “relationship with other variables,” as one of the five sources of validity evidence, will strengthen the DREEM [60]. In addition, as mentioned earlier, most reported DREEM scores fall into the category “more positive than negative,” in which scores can vary by as much as 50 points. Investigating the relationship between the DREEM and other variables will help establish the practical meaning of differences in DREEM scores that are masked within that category or offered as mere numbers.

## Conclusion

To understand the relationship between DREEM scores and the subjective happiness of students in a single medical school, we first analyzed the scores according to students’ phase in the curriculum and personal characteristics, and then we examined the factors that influenced happiness. Significant differences in the DREEM scores over time were identified, whereas no difference was identified for subjective happiness. The effects of GPA on happiness disappeared when other variables were controlled; only the influence of the SSSP remained significantly positive among the five subscales. Putting these results together, our study suggests that institutions need to work on using an integrated approach throughout their curriculum and creating an appropriate educational environment that promotes not only students’ academic development but also their personal development and social relationships.

## Data Availability

Data and materials used during the study are available from the corresponding author on reasonable request.

## Abbreviations

CBME: Competency-Based Medical Education
DREEM: Dundee Ready Educational Environment Measure
EUSOM: Eulji University School of Medicine
GPA: Grade Point Averages
QOL: Quality of Life
SASP: Students’ Academic Self-Perceptions
SPA: Students’ Perceptions of Atmosphere
SPL: Students’ Perception of Learning
SPP: Students’ Academic Self-Perceptions
SPT: Students’ Perception of Teaching
SSSP: Students’ Social Self-Perceptions
UME: Undergraduate Medical Education
